# NaCl Concentration-Dependent Aminoglycoside Resistance of *Halomonas socia* CKY01 and Identification of Related Genes

**DOI:** 10.4014/jmb.2009.09017

**Published:** 2020-11-04

**Authors:** Ye-Lim Park, Tae-Rim Choi, Hyun Joong Kim, Hun-Suk Song, Hye Soo Lee, Sol Lee Park, Sun Mi Lee, Sang Hyun Kim, Serom Park, Shashi Kant Bhatia, Ranjit Gurav, Changmin Sung, Seung-Oh Seo, Yung-Hun Yang

**Affiliations:** 1 Department of Biological Engineering, College of Engineering, Konkuk University, Seoul 05029, Republic of Korea; 2 Institute for Ubiquitous Information Technology and Applications (CBRU), Konkuk University, Seoul 0509, Republic of Korea; 3 Doping Control Center, Korea Institute of Science and Technology, Seoul 02792, Republic of Korea; 4 Department of Food Science and Nutrition, The Catholic University of Korea, Bucheon 1662, Republic of Korea

**Keywords:** *Halomonas* strain, antibiotic resistance, salt tolerance, genome sequencing

## Abstract

Among various species of marine bacteria, those belonging to the genus *Halomonas* have several promising applications and have been studied well. However, not much information has been available on their antibiotic resistance. In our efforts to learn about the antibiotic resistance of strain *Halomonas socia* CKY01, which showed production of various hydrolases and growth promotion by osmolytes in previous study, we found that it exhibited resistance to multiple antibiotics including kanamycin, ampicillin, oxacillin, carbenicillin, gentamicin, apramycin, tetracycline, and spectinomycin. However, the *H. socia* CKY01 resistance pattern to kanamycin, gentamicin, apramycin, tetracycline, and spectinomycin differed in the presence of 10% NaCl and 1% NaCl in the culture medium. To determine the mechanism underlying this NaCl concentration-dependent antibiotic resistance, we compared four aminoglycoside resistance genes under different salt conditions while also performing time-dependent reverse transcription PCR. We found that the *aph2* gene encoding aminoglycoside phosphotransferase showed increased expression under the 10% rather than 1% NaCl conditions. When these genes were overexpressed in an *Escherichia coli* strain, pETDuet-1::*aph2* showed a smaller inhibition zone in the presence of kanamycin, gentamicin, and apramycin than the respective control, suggesting *aph2* was involved in aminoglycoside resistance. Our results demonstrated a more direct link between NaCl and aminoglycoside resistance exhibited by the *H. socia* CKY01 strain.

## Introduction

Many halotolerant microorganisms are easily found in marine ecosystems [1-3]. The marine environment provides ample resources from terrestrial microbes such as hydrolytic enzymes, biopolymers, including exopolysaccharides and polyhydroxyalkanoates (PHAs), and also for producing osmolytes that stabilize biomolecules and cellular structure [4-7]. Among the various species of marine bacteria, those belonging to the genus *Halomonas* have promising biotechnological applications [8, 9]. In our previous study, we screened out hyper- producer of poly 3-hydroxyalkanoates (PHB) *Halomonas* sp. YLGW01 and various hydrolase producer *Halomonas socia* CKY01 from marine soil in South Korea, and performed genomic sequencing of *H. socia* CKY01 [10-12].

Although resistance to multiple antibiotics under different environmental conditions exhibited by *Halomonas* strains is not an immediate threat to human health because *Halomonas* is not a well-known pathogen, and the antibiotic resistance exhibited by some species belonging to this genus has not threatened human life so far, antibiotic resistance in this species of bacteria could become a serious health problem in the future [13, 14]. Specifically, transient or hidden antibiotic resistance noticed in these marine bacteria could result in unexpected issues related to bacterial growth control. Considering our proximity to the marine environment, these antibiotic-resistant microorganisms could easily spread to our environment and present a critical danger for the food industry because high salt concentrations are used for food preservation [15, 16]. Combining their osmoprotection and antibiotic resistance, marine bacteria could survive in unusual conditions unlike terrestrial bacteria. Therefore, further studies on antibiotic resistance in marine bacteria and the effect of osmolytes on marine bacterial growth are needed when considering these bacteria, along with their advantages and disadvantages, as sources of compounds of interest.

Since *Halomonas* bacteria are regarded as environmentally friendly and are easily found in marine environments, previous studies on these bacteria did not focus on their antibiotic resistance traits, leading to only a few reports on antibiotic resistance by *Halomonas* bacteria. In our previous study, we also showed that the *H. socia* CKY01 strain contained various antibiotic resistance genes and exhibited β-lactam antibiotic resistance [12]. Unlike the studies performed to understand the pathogenicity of *Vibrio* and *Acinetobacter* species, antibiotic resistance issues and specific resistance mechanisms involving *Halomonas* species have not been researched in depth [16-18]. To explain NaCl-dependent antibiotic resistance, previous studies used the omics approach, describing the changes in redox state, differential expression of efflux pump genes, or cross-protection effect [16, 17, 19]. However, the mechanism by which the stress response program affects the susceptibility of these bacteria to antibiotic treatment remains unclear [19-22].

Therefore, we studied NaCl concentration-dependent aminoglycoside resistance, which we discovered for the first time in *H. socia* CKY01. We found that *H. socia* CKY01 showed NaCl concentration-dependent behavior and the presence of NaCl affected the expression of an aminoglycoside-modifying enzyme, aminoglycoside phosphotransferase (APH2), which seemed to play an important role in *H. socia* CKY01. Our results showed a direct link between the NaCl concentration and aminoglycoside resistance exhibited by this strain.

## Materials and Methods

### Chemicals

All chemicals used in the present study were of analytical grade or higher. Antibiotics such as kanamycin, chloramphenicol, ampicillin, oxacillin, carbenicillin, gentamicin, apramycin, tetracycline, and spectinomycin as well as proline were purchased from Sigma-Aldrich (USA). Other chemicals used in growth media were also purchased from Sigma-Aldrich.

### Growth Test Media and Culture Conditions

To confirm antibiotic resistance under different salt concentrations, *H. socia* CKY01 was cultured in Luria-Bertani (LB) medium supplemented with 1% or 10% NaCl and 50 μg/ml kanamycin, 35 μg/ml chloramphenicol, 100 μg/ml ampicillin, 6 μg/ml oxacillin, 100 μg/ml carbenicillin, 25 μg/ml gentamicin, 50 μg/ml apramycin, 0.5 μg/ml tetracycline, and 100 μg/ml spectinomycin, respectively. The appropriate concentrations of antibiotics were pre-tested and selected for growth comparison. Cultures were incubated at 30°C and 200 rpm in shaking incubator, and the optical density of cell growth was measured using a spectrometer at 600-nm wavelength every 24 to 72 h.

To test the growth of *H. socia* CKY01 in the presence of 50 μg/ml kanamycin in different salinity conditions, *H. socia* CKY01 cultures containing 1, 3, 5, 10, 13, 15, 17, and 20% NaCl with or without kanamycin in LB medium were incubated at 30°C and 200 rpm. Thereafter, cell growth was measured by comparing cell growth at 24 h and 48 h. For determining the effect of osmolytes on cell growth in the presence of kanamycin and at different NaCl concentrations, 60 mM proline was added along with 50 μg/ml kanamycin to LB medium and the cell growth was compared with that observed after adding kanamycin alone [12]. The incubation condition was the same as described above and all tests were duplicated.

### Reverse Transcription (RT)-PCR

*H. socia* CKY01 was grown for 48 h in LB medium containing 1% or 10% NaCl, and the cells were then treated with 50 μg/ml kanamycin. Total RNA was extracted at 1, 3, and 24 h after adding kanamycin by using the RNeasy Mini Kit (Qiagen, Germany) according to the manufacturer’s protocol. RNA purity was determined by performing 1% agarose gel electrophoresis and the quantity of total RNA was measured by using a NanoDrop system (Thermo Fisher Scientific, USA). The samples were diluted at the same concentration and used for first-strand cDNA synthesis, for which random hexamer primers were used along with the Invitrogen SuperScript III Kit (Thermo Fisher) according to the manufacturer’s instructions. Changes in the expression levels of different genes were compared with that of a *16S rRNA* gene used as a control by performing semi-quantitative RT-PCR. The PCR product concentrations were compared by performing 1% agarose gel electrophoresis.

### Construction of *Escherichia coli* Strains

Strain and plasmids used in this study are listed in Table 1. *E. coli* BL21 (DE3) was used as the host strain for gene cloning. The strains were cultured in lysogeny broth Luria-Bertani (LB) agar and liquid broth for cell preparation and selection of transformants. LB agar was composed of 10 g tryptone, 5 g yeast extract, 10 g NaCl, and 15 g agar in 1 l distilled water. Ampicillin (100 μg/ml) was added when required.

Gene cloning was performed according to standard protocols [23]. Four genes related to aminoglycoside resistance, namely aminoglycoside phosphotransferase (*aph1*), aminoglycoside phosphotransferase (*aph2*), *aac*-I family aminoglycoside N-acetyltransferase (*aac*), and aminoglycoside nucleotidyltransferase (*ant9*), were obtained from *H. socia* CKY01. The target genes were amplified by PCR and the products were purified for restriction enzyme digestion. The digestion products were ligated into a pETDuet-1 plasmid that was also digested with the same enzymes, followed by transformation into the *E. coli* BL21 (DE3) strain by using the heat-shock method [24, 25]. The constructed plasmids were confirmed by sequencing.

### Antimicrobial Susceptibility Test

*E. coli* BL21 containing the different plasmids was subject to the antimicrobial susceptibility test according to the disc diffusion assay [26] and *E. coli* BL21 containing pETDuet-1 was used as a control. To perform the disc diffusion assay, *E. coli* strains were pre-cultured in LB medium for 18 h at 37°C with shaking at 200 rpm in a shaking incubator. Then, an LB agar plate containing 100 μg/ml ampicillin and 0.05 mM isopropyl-β-D-thiogalactoside (IPTG) was prepared for protein expression. The strains were tested for resistance against the following antibiotics: kanamycin, apramycin, gentamicin, and tetracycline. The stock solutions of kanamycin, apramycin, and gentamicin had concentrations of 0.05, 0.1, 0.2, 0.3, 0.4, and 0.5 mg/ml. Paper discs (ADVANTEC, 6mm) were prepared each with 20 μl of the antibiotic stock solution and dried. The dried paper discs were placed on strain spread-agar plates incubated at 30°C for 24 h and the inhibition zone diameter was measured. The antibiotic content on the disc was calculated by multiplying the stock concentration by 20 μl.

## Results and Discussion

### Determining Antibiotic Resistance at High NaCl Concentration

**Fig. 1:**
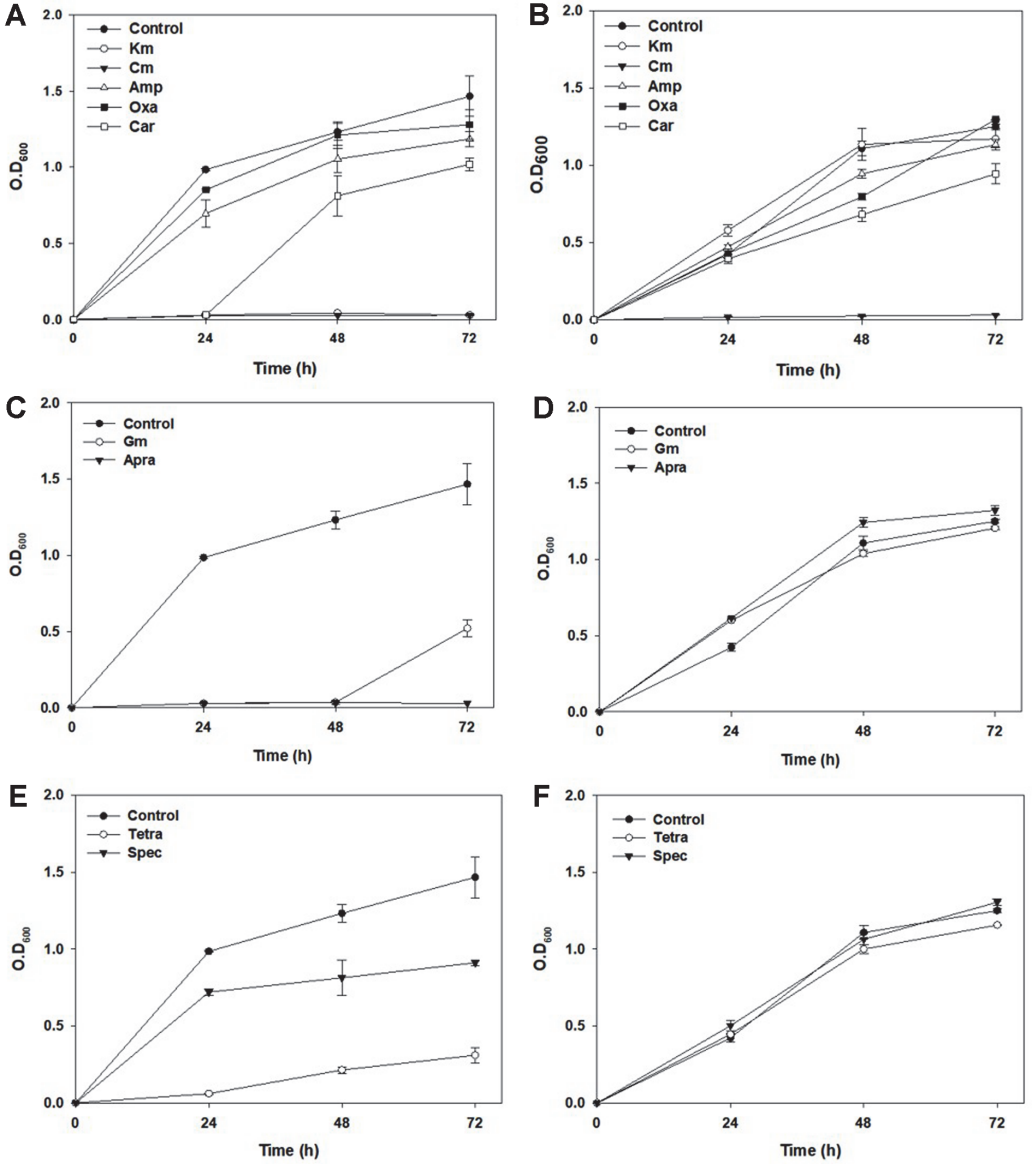
Antibiotic resistance test under different salinity conditions. (**A**) Growth observation in 1% NaCl after 72 h of cultivation at 30°C in the presence of 50 μg/ml kanamycin (Km), 35 μg/ml chloramphenicol (Cm), 100 μg/ml ampicillin (Amp), 6 μg/ml oxacillin (Oxa), or 100 μg/ml carbenicillin (Car). (**B**) Growth observed in the presence of 10% NaCl under the same conditions as (**A**). (**C**) Growth observed in the presence of 1% NaCl, 25 μg/ml gentamicin (Gm), and 50 μg/ml apramycin (Apra). (**D**) Growth observed in the presence of 10% NaCl and under the same conditions as (**C**). (**E**) Growth observed in the presence of 1% NaCl, 0.5 μg/ml tetracycline (Tetra), and 100 μg/ml spectinomycin (Spec). (**F**) Growth observed in the presence of 10% NaCl and under the same conditions as (**E**).

When performing initial characterization of *H. socia* CKY01, various antibiotics such as kanamycin, chloramphenicol, ampicillin, oxacillin, carbenicillin, gentamicin, apramycin, tetracycline, and spectinomycin were tested in the medium containing 1% and 10% NaCl. The strain showed antibiotic resistance to β-lactams such as 100 μg/ml ampicillin, 6 μg/ml oxacillin, and 100 μg/ml carbenicillin, but no resistance to 50 μg/ml kanamycin or 35 μg/ml chloramphenicol was observed when the cells were grown in a medium containing 1%NaCl for 24 h (Fig. 1A). When 10% NaCl-containing medium was used, *H. socia* CKY01 showed slightly decreased growth compared to use of 1% NaCl since high salt condition could effect and stress the bacteria growth [8] (Fig. 1B). Although the growth pattern of *H. socia* CKY01 under 10% NaCl was a little bit different, *H. socia* CKY01 showed a similar resistance pattern for all the antibiotics except kanamycin. However, the bacterium showed resistance to kanamycin when 10% NaCl was added to the medium, as opposed to the result obtained with 1% NaCl, which did not show any growth (Figs. 1A and 1B). *H. socia* CKY01 grew well with kanamycin under 10%NaCl like the control, which did not have kanamycin in the media. In a previous study on *Vibrio alginolyticus* [16], the minimum inhibitory concentration (MIC) increased as the NaCl concentration increased, especially when aminoglycoside antibiotics were used.

To confirm whether NaCl exerted a similar effect on *H. socia* CKY01 cells, we examined the effect of different aminoglycosides, specifically 25 μg/ml gentamicin and 100 μg/ml apramycin. We also examined the effects of 0.5 μg/ml tetracycline and 100 μg/ml spectinomycin, which are not aminoglycosides, but act similarly to treatment with aminoglycosides as they target both the 30S and 50S ribosomal subunits in the bacteria. We found that the cells did not show antibiotic resistance in the presence of gentamicin and apramycin at 1% NaCl concentration (Fig. 1C) but high antibiotic resistance was observed in the presence of kanamycin at 10% NaCl (Fig. 1D). The cells showed some resistance to both spectinomycin and tetracycline at 1% NaCl concentration (Fig. 1E), which is a little bit different from the results obtained for aminoglycosides; however, resistance to spectinomycin and tetracycline also increased in the presence of 10% NaCl (Fig. 1F). Although *V. alginolyticus* showed a different distribution of pyruvate cycle intermediates and reduction of redox states in the presence of NaCl that resulted in increased antibiotic resistance [16], less information on antibiotic resistance by *Halomonas* was reported that could explain a more direct link to aminoglycoside resistance in the previous study [18]. Although some undiscovered mechanisms pertaining to increased resistance to spectinomycin and tetracycline exist, we focused on determining the reason behind aminoglycoside resistance and studied NaCl concentration-dependent aminoglycoside resistance in the present study.

### NaCl Concentration-Dependent Kanamycin Resistance

**Fig. 2:**
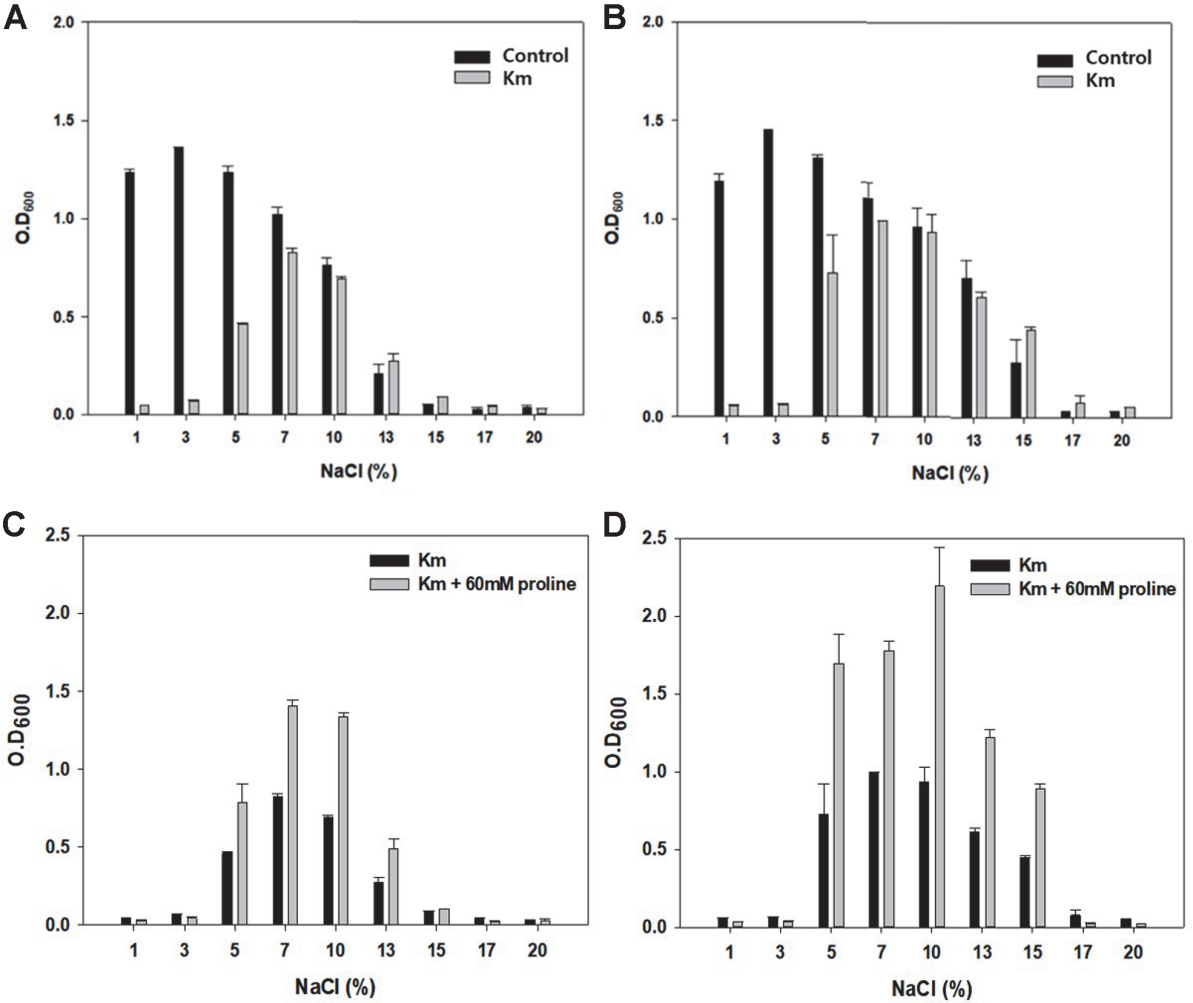
Growth of *H. socia* CKY01 after adding kanamycin and under different salinity conditions. (**A**) Growth observed with and without 50 μg/ml kanamycin (Km) after 24 h of cultivation. (**B**) Growth observed after 48 h under the same conditions as (**A**). (**C**) Growth observed with and without 60 mM proline in the presence of 50 μg/ml kanamycin after 24 h of cultivation. (**D**) Growth observed after 48 h under the same conditions as (**C**).

To determine the effect of salt concentration on kanamycin resistance, we treated the *H. socia* CKY01 cells to different concentrations of NaCl (from 1% to 20%) with and without kanamycin (Figs. 2A and 2B). The cells showed maximum growth at 3% NaCl and grew in the presence of up to 15% NaCl without kanamycin. When kanamycin was added to the media at the start of culture, the cells showed kanamycin resistance in the presence of 5% NaCl. The cells did not show any resistance at 1% and 3% NaCl but showed high resistance at 7% and 10% NaCl at 24 and 48 h. After 48 h, 7 to 10% NaCl led to high kanamycin resistance, resulting in no difference in growth with or without kanamycin at >7% NaCl concentration (Fig. 2B). Cell growth under the presence of different concentrations of NaCl clearly showed an increase in antibiotic resistance to kanamycin as the concentration of NaCl increased.

To determine whether osmolytes such as proline could increase antibiotic resistance, since it showed a protective effect in the presence of NaCl that resulted in an increased growth at high NaCl concentrations [12, 27, 28], 60 mM of proline was added to the growth medium (Figs. 2C and 2D). We found that proline increased cellular growth in the range of 5%-15% NaCl where the strain had shown kanamycin resistance. However, it did not increase antibiotic resistance at 1 to 3% NaCl concentration because there is no difference in the region that showed susceptibility and both the strains could not grow together. A certain metabolite level is required to influence antibiotic resistance because an osmolyte such as proline could protect the cells from the adverse effects of high salinity-related stress [12, 29, 30]. However, it could not enhance antibiotic resistance itself because resistance to antibiotics involves a different mechanism that is dependent on the concentration of salts such as NaCl.

Considering that cell surface hydrophobicity was decreased in several studies after exposure to different types of antibiotics, such as β-lactams and aminoglycosides [31], and high salinity could make the cell envelope more tightly packed and reduce permeability [32], we measured the hydrophobicity of *H. socia* CKY01. Considerably complex results were obtained, however, suggesting that there is no direct correlation between hydrophobicity and NaCl concentrations (data not shown).

### Analyzing the Expression of Kanamycin Resistance Genes 

Based on whole genome sequencing from previous research, we found three β-lactam resistance genes encoding the following enzymes: metallo-β-lactamase-like fold metallo-hydrolase (orf00605), class D β-lactamase (orf02848), and serine hydrolase (orf04462) (Table 2). Because the β-lactamase hydrolyses the amide bond in the β-lactam ring of β-lactam antibiotics such as penicillin and cephalosporin, the three genes explained the resistance to β-lactam antibiotics [33, 34]. However, the aminoglycoside resistance results of *H. socia* CKY01 were more interesting due to its NaCl-dependent resistance; therefore, we also identified four genes related to aminoglycoside resistance and targeted these for analysis as follows: aminoglycoside phosphotransferase (*aph1*, orf00432, 61.38% homology to aminoglycoside phosphotransferase from the *Halomonadaceae* bacterium (MAX33989.1)); aminoglycoside phosphotransferase (*aph2*, orf06107, 70.04% homology to aminoglycoside phosphotransferase from *Halomonas*. sp. (MAP35044.1)); *aac*-I family aminoglycoside N-acetyltransferase (*aac*, orf04314, 88.46% homology to AAC(3)-I family aminoglycoside N-acetyltransferase from *Halomonas* sp. 1513 (WP_076748323.1)); and aminoglycoside nucleotidyltransferase (*ant*, orf03824, 59.11% homology to aminoglycoside nucleotidyltransferase ANT9 from *Ectothiorhodospira* sp. PHS-1 (WP_008931083.1)) (Table 2). There are three routes for inactivation of aminoglycoside antibiotics: ATP-dependent O-phosphorylation by aminoglycoside phosphotransferase (APH), acetyl CoA-dependent N-acetylation by aminoglycoside N-acetyltransferase (AAC) and ATP-dependent O-adenylation by aminoglycoside nucleotidyltransferase (ANT), thus, the genes from *H. socia* CKY01 were expected to exhibit the aminoglycoside modification function based on annotation and BLAST results [35].

Considering *H. socia* CKY01 has at least four aminoglycoside resistance-related genes located at different genomic loci (Table 2), we expected the cells to show kanamycin resistance, but in the presence of 1% NaCl, the cells did not exhibit any antibiotic resistance as mentioned earlier. Although there are several reasons for antibiotic resistance, such as differing distribution of pyruvate cycle intermediates and changes in redox states caused by NaCl, membrane fluidity, and hydrophobicity caused by the presence of high concentrations of salt [16, 36, 37], there has been no report on the direct effect of resistance genes in the presence of NaCl. Based on antibiotic susceptibility at 1% NaCl, we could not expect a simultaneous shutdown of these antibiotic resistance genes altogether. Hence, we sought to determine any changes in the expression of the aminoglycoside resistance genes in the presence of 1% to 10% NaCl.

**Fig. 3:**
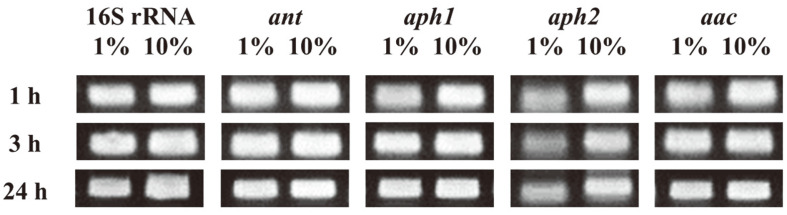
Time-dependent semi-RT PCR expression results. The gene expressions of kanamycin resistance-related genes of *H. socia* CKY01 were observed after adding kanamycin to LB medium containing 1% and 10% NaCl after culturing for 48 h.

Based on the genetic information available for the four aminoglycoside resistance-related genes, we obtained mRNA expression data in the presence of different NaCl concentrations (1% and 10%). The cells were treated with 1% and 10% NaCl for 48 h. Then, the cultured cells were treated with kanamycin and total RNA was extracted after 1, 3, and 24 h, and this was used to prepare cDNA as explained in the Materials and Methods. We compared the mRNA expression of the 16S rRNA gene as control, as well as that of ant, *aph1*, *aph2*, and *aac* genes by RT-PCR (Fig. 3). When we compared the results obtained after 1% and 10% NaCl treatment, there was no considerable difference in the expression of *ant* and *aac*; however, *aph1* and *aph2* seemed to be more expressed under 10% NaCl concentration at 1 h. After 3 h, only *aph2* showed a distinct difference suggesting that *aph2* was differently overexpressed at NaCl concentrations ranging between 1% and 10%. As mentioned earlier, although there should be many genes involved in NaCl-related antibiotic resistance, and the RT-PCR showed the relative expression levels at different time periods and NaCl concentrations and does not suggest the actual expression levels of all the genes in vivo, our result showed that *aph2* responded differently to various concentrations of NaCl, causing NaCl concentration-dependent resistance to aminoglycosides and thus providing more direct evidence of the aminoglycoside resistance exhibited by these cells.

### Overexpression of Aminoglycoside Resistance Genes

To determine the function of each gene introduced in *E. coli* BL21, we amplified the four genes by PCR and cloned them in the pETDuet-1 vector (Table 1). Although deletion of each gene in *H. socia* CKY01 seemed the best way to reveal their individual function, this is a newly identified *Halomonas* strain and currently, there are no molecular biology tools available for *H. socia* CKY01 so far. Future molecular biology-based studies could therefore be helpful in performing deletion experiments.

**Fig. 4:**
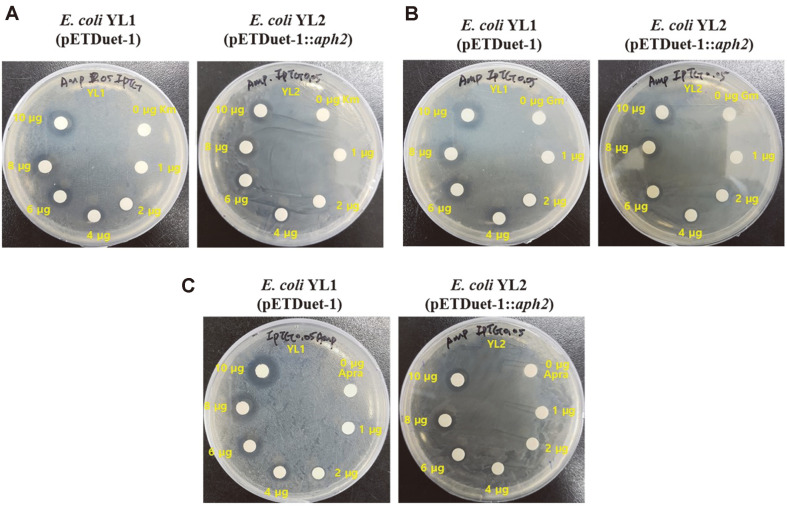
Results of the MIC test performed by treating constructed *E. coli* strains with kanamycin, gentamicin and apramycin. (**A**) Kanamycin MIC result of *E. coli* strains after 24 h cultivation at 30°C in the presence of 100 μg/ml ampicillin and 0.05 mM IPTG. Paper discs contained 0, 1, 2, 4, 6, 8, and 10 μg kanamycin. (**B**) Gentamicin MIC result of *E. coli* strains under the same conditions as (**A**). (**C**) Apramycin MIC result of *E. coli* strains under the same conditions as (**A**).

Although the expression levels of these genes were very low in the SDS-PAGE test (data not shown), to determine the function of each gene, we grew *E. coli* cells in LB for 18 h and then the same quantity of cells was spread on an LB plate containing 0.05 mM IPTG. Using a paper disc, we determined the gene functions at different concentrations of kanamycin. Initially, to determine the optimal concentration of kanamycin, we used 200 μg to 1,000 μg of the antibiotic but the cells did not show resistance with this concentration range (data not shown). Hence, we used 1 to 10 μg kanamycin. Both *E. coli* YL1 and *E. coli* YL2 survived in the presence of 1 μg and 2 μg kanamycin, showing the biggest inhibition zone size at 10 μg, and different inhibition zone sizes in 4 μg kanamycin (Fig. 4A). The *E. coli* YL1 strain containing pETDuet-1 developed an inhibition zone in the presence of 4 μg kanamycin measuring 9.5 ± 0.5 mm, while the *E. coli* YL2 strain containing pETduet::*aph2* developed a smaller zone diameter in the presence of 4 μg kanamycin at 7.5 ± 0.5 mm (Table 3). When we treated these strains with gentamicin, they had resistance in the presence of 1 μg and 2 μg gentamicin and showed different size inhibition zones with 4 μg kanamycin (Fig. 4B). The *E. coli* YL1 strain developed an inhibition zone measuring 9.5 ± 0.5 mm, while *E. coli* YL2 developed a smaller inhibition zone of 6.8 ± 0.3 mm, similar to that seen after kanamycin treatment (Table 3). Also, we treated two strains with apramycin, and *E. coli* YL1 developed an inhibition zone from 4 μg, whereas *E. coli* YL2 developed an inhibition zone from 6 μg apramycin (Fig. 4C). The inhibition zone size of *E. coli* YL1 was 10.5 ± 0.3 mm in the presence of 6 μg apramycin, while *E. coli* YL2 developed an inhibition zone of 7.3 ± 0.3 mm in the presence of 6 μg apramycin (Table 3). When we compared the size of the inhibition zones with the same concentrations of antibiotics, the diameter of the inhibition zone developed by *E. coli* YL2 was smaller than that of *E. coli* YL1. Although the difference was not considerable, *E. coli* YL2 containing *aph2* clearly showed more resistance to aminoglycoside antibiotics than the control.

The inhibition data obtained using *E. coli* might be different from that obtained using *H. socia* CKY01 because of the difference in the expression levels of these genes in vivo, and other antibiotic resistance genes such as *aac*, *aph*, and *ant* present in *E. coli* did not cause distinct resistance (data not shown). However, our results showed that *H. socia* CKY01 exhibited antibiotic resistance wherein the expression level of *aph2* increased in the presence of 10% NaCl. Thus, *aph2* is an important gene that confers aminoglycoside resistance to this bacterial strain wherein the differential expression of this gene at different NaCl concentrations directly affects the antibiotic resistance capability of *H. socia* CKY01.

## Conclusions

Although marine bacteria are useful sources of various compounds, the antibiotic resistance exhibited by these bacteria has not been discussed in as much detail as that for pathogens such as *Vibrio* and *Acinetobacter* [16, 17]. By performing antibiotic resistance assays and genomic sequencing, we identified that *H. socia* CKY01 is resistant to various antibiotics as it carries antibiotic resistance genes. For example, it has at least three β-lactamase genes that confer resistance to ampicillin and oxacillin, and four aminoglycoside resistance genes against kanamycin. Interestingly, these genes do not confer permanent, but only transient antibiotic resistance under different stress conditions. For example, *H. socia* CKY01 showed different levels of resistance specifically against kanamycin in the presence of 1% and 10% NaCl. Therefore, by controlling the expression of the four identified aminoglycoside resistance genes, our study showed *H. socia* CKY01 exhibited NaCl concentration-dependent resistance and even demonstrated a more direct link between aminoglycoside resistance and NaCl concentration. Although further study into the detailed mechanisms of player-like regulatory proteins is required to understand how this transient expression happens, our results demonstrated that the activities of the antibiotic-modifying enzymes present in this strain are dependent on NaCl concentration. We believe that a decrease of antibiotic resistance at high NaCl concentrations might be another interesting topic for future studies and our work has provided a new avenue in the form of transient antibiotic resistance exhibited by *H. socia* CKY01.

## Figures and Tables

**Table 1:** Bacterial strains and plasmids used in this study.

Strain or plasmid	Description	Reference
Strains		
*E. coli* BL21(DE3)	Expression host	Invitrogen
*E. coli* YL1	*E. coli* BL21(DE3) harboring pETDuet-1	This study
*E. coli* YL2	*E. coli* BL21(DE3) harboring pETDuet-1::*aph2*	This study
Plasmids		
pETDuet-1	pT7, PBR322 ori, Amp^r^	[38]
pETDuet-1::*aac*	pETDuet-1 containing *aac* from *H. socia* CKY01	This study
pETDuet-1:: *aph1*	pETDuet-1 containing *aph1* from *H. socia* CKY01	This study
pETDuet-1::*aph2*	pETDuet-1 containing *aph2* from *H. socia* CKY01	This study
pETDuet-1::*ant*	pETDuet-1 containing *ant* from *H. socia* CKY01	This study

**Table 2:** List of antibiotic resistance-related genes in *Halomonas socia* CKY01.

Antibiotic resistance	Sequence name	Sequence description	Gene ontologies
β-lactam antibiotics	orf00605	Metallo-β-lactamase-like fold metallo-hydrolase	P: antibiotic catabolic process; F: zinc ion binding; F: beta-lactamase activity; P: penicillin biosynthetic process
	orf02848	class d beta-lactamase	F: penicillin binding; F: beta-lactamase activity; P: antibiotic catabolic process
	orf04462	serine hydrolase	P: beta-lactam antibiotic catabolic process; F: beta-lactamase activity; P: response to antibiotic; F: hydrolase activity
Aminoglycoside antibiotics	orf00432	aminoglycoside phosphotransferase (*aph1*)	P: metabolic process; F: transferase activity, transferring phosphorus-containing groups
	orf06107	aminoglycoside phosphotransferase (*aph2*)	P: metabolic process; F: transferase activity, transferring phosphorus-containing groups
	orf04314	*aac*-I family aminoglycoside n-acetyltransferase (*aac*)	F: N-acetyltransferase activity; P: acyl-carrier-protein biosynthetic process
	orf03824	aminoglycoside nucleotidyltransferase (*ant*)	F: nucleotidyltransferase activity; F: nucleotide binding

*P: biological process, F:molecular function

**Table 3:** Results of the MIC test using the constructed *E. coli* strains.

Antibiotics	Strain	Antibiotic on disc (μg)	Inhibition zone diameter (mm)
Kanamycin	YL1 (pETduet-1)	4	9.5 ± 0.5
	YL2 (pETduet-1::a*ph2*)	4	7.5 ± 0.5
Gentamicin	YL1 (pETduet-1)	4	9.5 ± 0.5
	YL2 (pETduet-1::a*ph2*)	4	6.8 ± 0.3
Apramycin	YL1 (pETduet-1)	6	10.5 ± 0.3
	YL2 (pETduet-1::a*ph2*)	6	7.3 ± 0.3
